# Gating at the Mouth of the Acetylcholine Receptor Channel: Energetic Consequences of Mutations in the αM2-Cap

**DOI:** 10.1371/journal.pone.0002515

**Published:** 2008-06-25

**Authors:** Pallavi A. Bafna, Prasad G. Purohit, Anthony Auerbach

**Affiliations:** Department of Biophysics and Physiology, State University of New York at Buffalo, Buffalo, New York, United States of America; Vrije Universiteit Amsterdam, Netherlands

## Abstract

Gating of nicotinic acetylcholine receptors from a **C**(losed) to an **O**(pen) conformation is the initial event in the postsynaptic signaling cascade at the vertebrate nerve-muscle junction. Studies of receptor structure and function show that many residues in this large, five-subunit membrane protein contribute to the energy difference between **C** and **O**. Of special interest are amino acids located at the two transmitter binding sites and in the narrow region of the channel, where **C**↔**O** gating motions generate a low↔high change in the affinity for agonists and in the ionic conductance, respectively. We have measured the energy changes and relative timing of gating movements for residues that lie between these two locations, in the C-terminus of the pore-lining M2 helix of the α subunit (‘αM2-cap’). This region contains a binding site for non-competitive inhibitors and a charged ring that influences the conductance of the open pore. αM2-cap mutations have large effects on gating but much smaller effects on agonist binding, channel conductance, channel block and desensitization. Three αM2-cap residues (αI260, αP265 and αS268) appear to move at the outset of channel-opening, about at the same time as those at the transmitter binding site. The results suggest that the αM2-cap changes its secondary structure to link gating motions in the extracellular domain with those in the channel that regulate ionic conductance.

## Introduction

**Figure 1:**
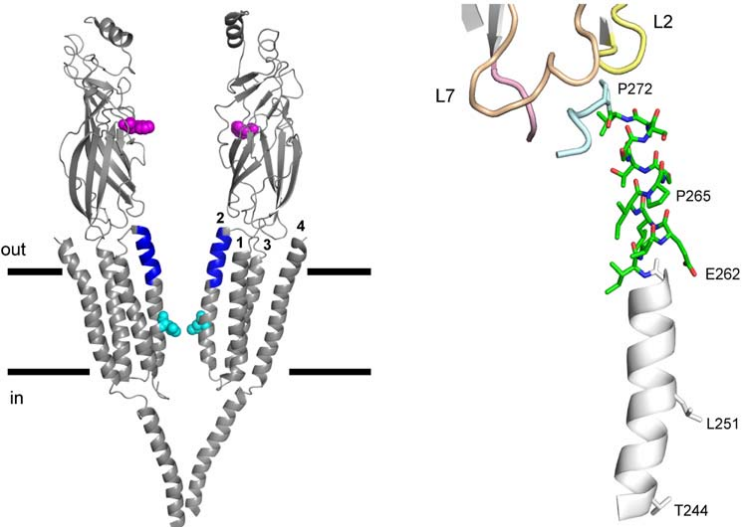
Structure of the αM2-cap in closed-unliganded *Torpedo* AChRs (PDB code 2bg9). *Left*, side view of the AChR (lines mark the lipid bilayer, ∼30 Å). The M2-cap domain in each of the two α subunits is blue; the two transmitter binding sites (αW149) are pink and the M2 equator residues (αL251, 9′) are cyan. The non-α subunits have been removed for clarity. The four membrane helices in the α_ε_ subunit are labeled: M2 lines the channel and M4 is at the periphery. *Right*, M2 and the M2-cap (residues 260–270) in the α_δ_ subunit (green, carbon; blue, nitrogen; red, oxygen). The ion permeation pathway is to the right, and helices M3 and M1 (not shown) are immediately to the left of M2. The M2-M3 linker (light blue), loop 2 (L2; yellow), loop 7 (L7, the ‘cys-loop’; tan) and the pre-M1 linker (pink) are near the cap. In M2 αE262 contributes to an ‘outer’ ring of charge, αL251 is the equator and αT244 forms a selectivity filter. Conserved proline residues in the M2-M3 linker (αP272) and in the M2-cap (αP265) are also labeled.

In the acetylcholine receptor-channel (AChR), the M2-cap lies at the junction of the extracellular vestibule and the narrow region of the ion permeation pathway (Fig. 1). In the mouse α subunit, the αM2-cap sequence is IVELIPSTSSA (residues 260–270; Table 1). There is a 4 Å cryo-EM structure of closed and unliganded *Torpedo* AChRs [1], a 1.94 Å resolution x-ray structure of a toxin-bound fragment of the mouse α subunit [2], and a 3.3 Å resolution structure of a prokaryotic member of the pentameric, ligand-gated channel superfamily [3]. However, as yet there are no high resolution structures of an intact AChR in either end state of the fully-liganded gating reaction, **A_2_C** or **A_2_O** (where **A** is the agonist). Here we report the channel opening (k_o_) and closing (k_c_) rate constants for 64 different mutations of nine αM2-cap residues in the mouse neuromuscular AChR (αI260-αS268), as well as the effects of these mutations on channel conductance, channel blockade and an approximate rate constant for entry into long-lived desensitized states.

**Table 1:** M2-cap Sequence Alignment of Cys-loop receptors.

subunit	18′	19′	20′	21′	22′	23′	24′	25′	26′	27′	28′
AChR α1	I	V	E	L	I	**P** ^265^	S	T	S	S	A
AChR α2	I	T	E	I	I	**P** ^276^	S	T	S	L	V
AChR α3	I	T	E	T	I	**P** ^269^	S	T	S	L	V
AChR α4	I	T	E	I	I	**P** ^279^	S	T	S	L	V
AChR α5	I	T	E	T	I	**P**	S	T	S	L	V
AChR α6	I	T	E	T	I	**P** ^274^	S	T	S	L	V
AChR α7	V	A	E	I	M	**P** ^264^	A	T	S	D	S
AChR α9	V	A	E	I	M	**P** ^102^	A	S	E	N	V
AChR α10	L	A	E	S	M	**P** ^270^	P	A	E	S	V
AChR β1	L	A	D	K	V	**P** ^276^	E	T	S	L	A
AChR β2	I	S	K	I	V	**P** ^265^	P	T	S	L	D
AChR β3	I	E	E	I	I	**P** ^270^	S	S	S	K	V
AChR β4	I	S	K	I	V	**P** ^262^	P	T	S	L	D
AChR δ	I	S	K	R	L	**P** ^279^	A	T	S	M	A
AChR γ	V	A	K	K	V	**P** ^274^	E	T	S	O	A
AChR ε	I	A	Q	K	I	**P** ^275^	E	T	S	L	S
GABA α1	A	R	N	S	L	**P** ^304^	K	V	A	Y	A
GABA α2	A	R	N	S	L	**P** ^305^	K	V	A	Y	A
GABA α3	A	R	N	S	L	**P** ^330^	K	V	A	Y	A
GABA α4	A	R	H	S	L	**P** ^337^	K	V	S	Y	A
GABA α6	A	R	H	S	L	**P** ^284^	K	V	S	Y	A
GABA β1	L	R	E	T	L	**P** ^298^	K	I	P	Y	V
Gly α1	S	R	A	S	L	**P** ^299^	K	V	S	Y	V
Gly β	L	A	A	E	L	**P** ^321^	K	V	S	Y	V
5HT 3_A_	V	S	D	T	L	**P** ^278^	A	T	A	I	G
5HT 3_B_	M	S	D	E	V	**P** ^289^	R	S	A	G	C

The entire sequence for the AChR α1 subunit (IVELIPSTSSA) is conserved in all vertebrates. Position 23′ is a proline (bold) in all cys-loop receptors. The superscripts on the conserved Pro represent the residue number.

**Table 2:** K_eq_ and Φ for αM2 and the αM2-M3 linker.

residue	domain	n mutants	Φ	±s.e.m	fold-change in K_eq_
T244 (2′)	M2 (filter)	3	-	-	1
L245 (3′)	M2	2	0.58	0.03	31
S246 (4′)	M2	7	0.67	0.17	140
I247 (5′)	M2	3	-	-	1
S248 (6′)	M2	2	0.67	0.14	8
V249 (7′)	M2	4	0.52	0.05	66
L250 (8′)	M2	3	0.63	0.02	7
L251 (9′)	M2 (gate)	5	0.26	0.04	740
S252 (10′)	M2	3	-	-	1
L253 (11′)	M2	5	0.55	0.08	211
T254 (12′)	M2	4	0.35	0.08	687
V255 (13′)	M2	3	0.51	0.01	11217
F256 (14′)	M2	3	0.72	0.06	291
L257 (15′)	M2	3	0.68	0.09	541
L258 (16′)	M2	4	0.59	0.13	271
V259 (17′)	M2	6	0.63	0.18	75
I260 (18′)	M2 CAP	5	0.89	0.04	333
V261 (19′)	M2 CAP	6	0.78	0.11	1000
E262 (20′)	M2 CAP (ring)	9	0.82	0.15	121
L263 (21′)	M2 CAP	9	0.66	0.12	250
I264 (22′)	M2 CAP	7	0.78	0.15	2547
P265 (23′)	M2 CAP	5	0.90	0.10	10250
S266 (24′)	M2 CAP	6	0.64	0.13	1000
T267 (25′)	M2 CAP	3	0.71	0.09	125
S268 (26′)	M2 CAP	6	0.97	0.11	4873
S269 (27′)	M2 CAP	3	0.65	0.06	358
A270 (28′)	M2 CAP	3	0.65	0.07	150
V271	M2-M3 Linker	4	-	-	1
P272	M2-M3 Linker	3	0.62	0.05	11850
L273	M2-M3 Linker	3	-	-	1
I274	M2-M3 Linker	4	0.62	0.04	2014
G275	M2-M3 Linker	3	0.65	0.06	88
K276	M2-M3 Linker	4	-	-	5

Residues αT244–αL258 from [5]; αV259 and αS269 from [38] ; αA270–αK276 from [18]

Estimates of the energetic consequences of individual side chain movements can be gained from measuring mutation-induced changes in the diliganded gating equilibrium constant (K_eq_), which is the which is the ratio k_o_/k_c_. K_eq_ depends on the difference in free energy between the entire protein in the **C** vs. **O** conformation. Therefore, a change in K_eq_ consequent to a mutation indicates that the perturbation caused the AChR to change this free energy difference, and, hence, the relative structure or dynamics (entropy) in the vicinity of the mutation, in the **A_2_C**↔**A_2_O** reaction. The extent to which a change in K_eq_ is determined by a change in k_o_ vs. k_c_ (given by the parameter Φ) may reflect mutation-induced changes in the transmission coefficient of the reaction [4], in which case Φ is a measure of the relative time within the reaction when the perturbed side chain flips from a **C**-like to an **O**-like conformation [5], [6].

The information regarding changes in energy and the transmission coefficient (K_eq_ and Φ, respectively) can be mapped onto the available structures to generate a framework for understanding AChR gating. These parameters (derived from experimental measurements of k_o_ and k_c_) have been estimated for dozens of residues (hundreds of mutations) in the adult form of the mouse neuromuscular AChR. At most positions, at least one side chain substitution causes a substantial change in K_eq_, with the majority of these sensitive sites residing in the α subunit and falling between the transmitter binding site (TBS) and the cytoplasmic limit of the transmembrane domain (TMD). In the extracellular domain (ECD) of the α subunit, the ‘moving’ residues are located mainly along the “+” side of the subunit interface (adjacent to either the δ or ε subunit) as well as throughout the interface with the TMD. In the TMD of the α subunit, at least one residue in all four membrane spanning helices is mutation-sensitive, including most of those in M2. These results suggest that the energy changes realized in gating are widespread, with no one structural transition standing out as being the single ‘on-off switch’ that separates **A_2_C** from **A_2_O**. With regard to Φ, values are clustered into domains that, as a first approximation, follow a coarse-grained and decreasing gradient along the long axis of the protein. This pattern suggests that the overall framework for the gating mechanism is that of an approximately linear sequence of stochastic domain motions (a ‘Brownian conformational wave’) that connects structural changes that regulate transmitter affinity with those that regulate conductance [7]. However, as described below, the timing of the αM2-cap gating motions do not neatly fit this pattern.

The M2-cap contains a high affinity binding site for non-competitive inhibitors (NCIs) that stabilize **D**(esensitized) conformations of the AChR, where the affinity for agonists is high (like in **O**) but the conductance of the channel is essentially zero (like in **C**) [8], [9]. Some NCIs have a high affinity specifically for **D** AChRs, while others may also act as traditional channel blockers that bind to the open pore [10], [11], [12]. A second function of the M2-cap is to regulate ionic conductance. All cys-loop receptors have a charged residue (opposite sign of the conducting ion) in the M2-cap (Table 1) [13], [14], [15]. More generally, disulfide-trapping experiments in GABA_A_ receptors [16] indicate that the upper portion of the M2 helix is flexible and dynamic because there is a fast rate of disulfide formation at two positions in the M2-cap of the α subunit (which corresponds to a non-α subunit in AChRs). Recently, Hilf and Dutzler [3] have suggested that channel-opening involves an outward tilt of the M2-cap domain.

Several AChR αM2-cap amino acids have previously been studied with respect to the effects of mutation on the kinetics of gating [17]. Mutations at positions α267–α269 significantly changed K_eq_ (indicating a gating motion) mainly by changing the channel-opening rate constant, but had little or no effect on the equilibrium dissociation constant for agonist binding to the **C** conformation (K_d_). Φ and changes in K_eq_ and K_d_ have also been estimated for the M2-M3 linker (α270–α276) [18]. Forman et al. [19] studied mutants of αE262 by using a combination of photo-modification (by 3-azioctanol) and fast patch perfusion. Most constructs decreased the EC_50_ for Ach, possibly by increasing K_eq_.

The results presented below show that αM2-cap residues have higher Φ-values than do the flanking residues in αM2, the αM2-M3 linker and loop 2. This pattern is discussed with respect to the overall framework for AChR gating and the conformational changes occurring at the mouth of the channel in the gating isomerization.

## Results

For alignment purposes, the amino acids of the entire M2 helix can be numbered sequentially from N- to C-terminus (intracellular-to extracellular, 1′–28′; M243-A270 in α subunit). Table 1 shows an alignment for the M2-cap (18′–28′) for all mouse AChR subunits plus representative subunits of other ‘Cys-loop’ receptors. Position 20′ is the outer charged ring of the pore and is an E in all AChR α subunits. Position 23′ is a completely-conserved P in all subunits of all Cys-loop receptors.

**Figure 2:**
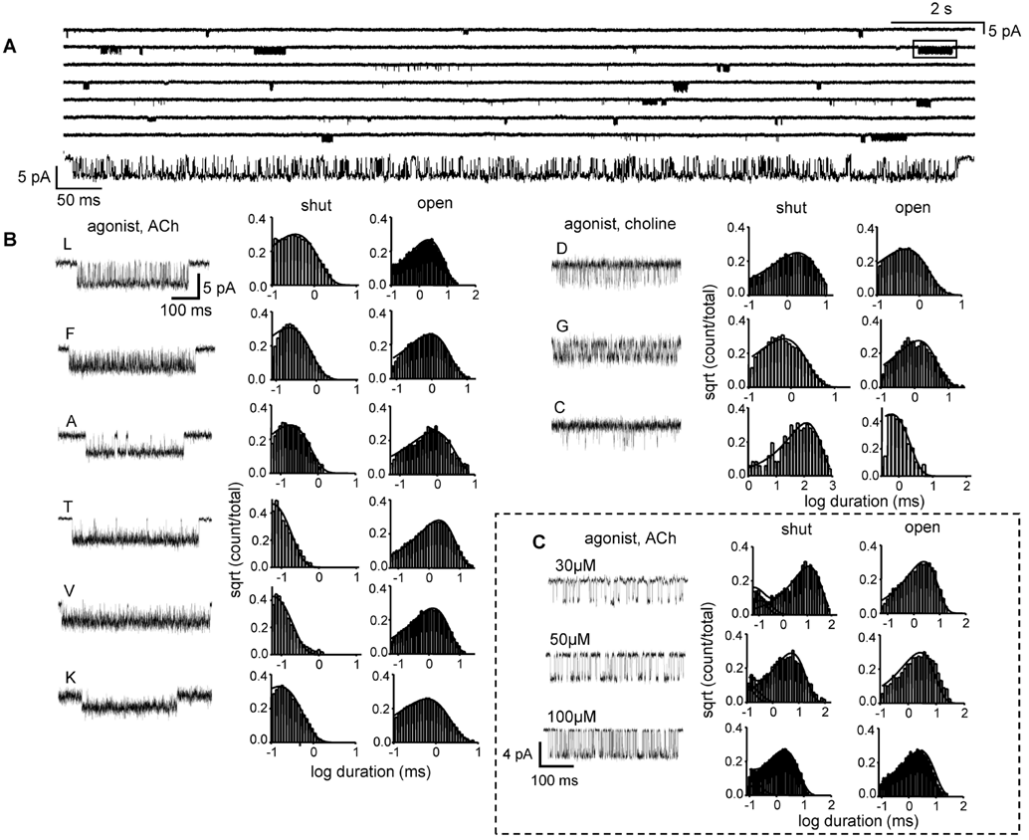
An example single-channel kinetic analyses (residue αE262; 20′). (A) Low time-resolution view of a continuous current trace for the mutant αE262L activated by 500 µM ACh (opening is down). Expanded view of boxed cluster shown, below. The long shut periods between clusters of openings represent desensitized AChRs. (B) Example clusters and interval duration histograms of 9 different αE262 mutations. Loss-of-function mutants (L, F, A, T, V and K) were activated by 500 µM ACh and gain-of-function mutants (D, G and C) were activated by 20 mM choline. Note the small single-channel current amplitude for the αE262K construct. (C) Estimation of ACh binding and gating rate constants in αE262L. Example clusters and shut/open interval duration histograms from AChRs activated by ACh. The solid lines are calculated from the rate constants obtained from the globally-optimized rate constants for all three patches (number of intervals: 30 µM, 2,336; 50 µM, 2,978; 100 µM, 8,631). There is no significant effect of this mutation on ACh binding to closed AChRs.

Fig. 1 shows the structure of the αM2-cap, based on the 4 Å cryo-EM model of closed, unliganded *Torpedo* AChRs (2bg9.pdb) [1]. Fig. 2 shows an example analysis for one position. Figure S1 displays example single-channel currents for all of the constructs. Tables S1, S2, S3 give the results in numeric form for the rate constant-, conductance-, channel block (by agonist)- and desensitization analyses.

**Figure 3:**
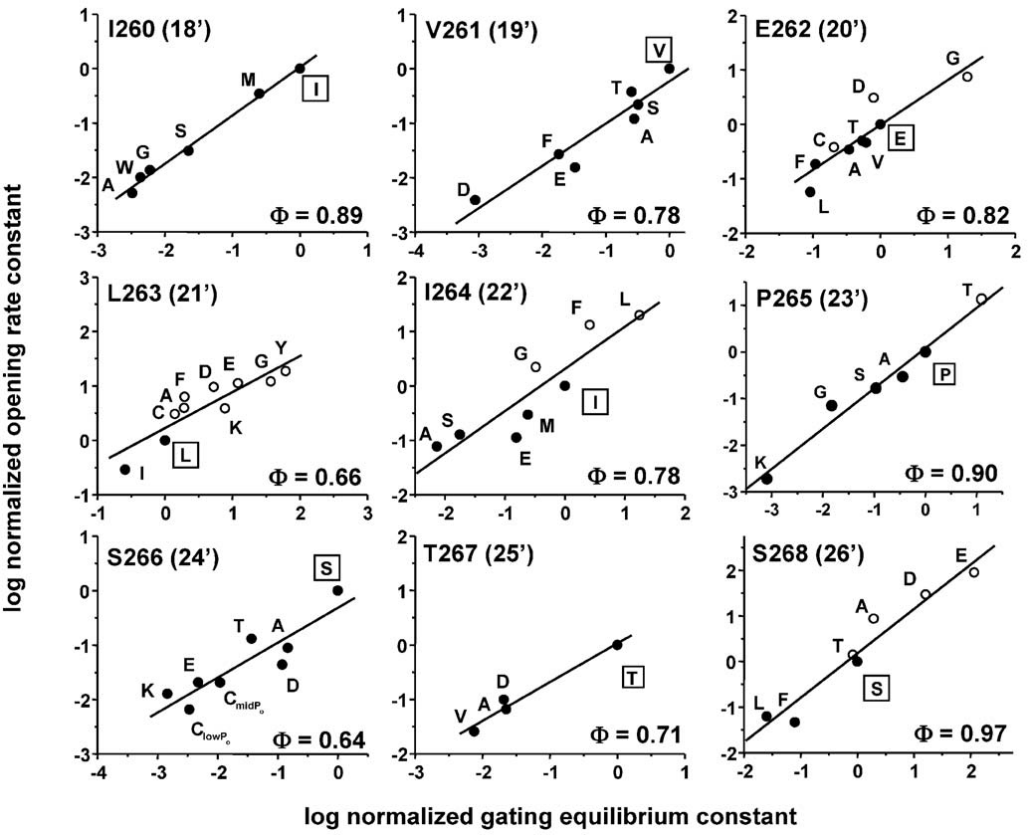
REFERs of αM2-cap residues. In each rate-equilibrium free-energy relationship (REFER) (residues I260–S268; 18′–26′), a point is the average of one mutant construct (Table S1). Φ is the slope of REFER. The Φ values are given in Table 2 and shown as a map in Fig. 5B. The agonist was either ACh (solid circles) or choline (open circles).

At least one side chain substitution at each of the αM2-cap positions changed K_eq_ by >10-fold (Fig. 3 and Table 2). Indeed, of the 7 positions in αM2 and the αM2-M3 linker that show a ≥1000-fold change in K_eq_, 5 are in the αM2-cap, with the most sensitive residues being αP265 (23′) and αS268 (26′). This result indicates that side chains of the αM2-cap change their energy (structure, dynamics or both) significantly between C and O conformations.

At four cap positions [αI260 (18′), αV261 (19′), αS266 (24′) and αT267(25′)] all side chain substitutions decreased K_eq_, and at five positions [αE262 (20′), αL263 (21′), αI264 (22′), αP265 (23′) and αS268(26′)] substitutions either increased or decreased K_eq_. There was no striking correlation between side chain chemistry and the change in K_eq_ at any position. Note that G, A, S, T, and K side chains were tolerated at the conserved αP265.

For all positions, the cap mutations changed K_eq_ mainly by changing k_o_ (resulting in high Φ values). The average Φ value for the entire region (α260–α270), calculated from the Φ estimate for each residue, was 0.77±0.12 (mean±s.d.), which is somewhat higher than for the flanking regions, the M2-M3 linker (α272–α275; 0.63±0.02) and M2 13′–17′ (α255–α259; 0.63±0.08). Three cap residues had particularly high Φ values, αS268, αP265 and αI260 (0.92±0.04). This result suggests that the αM2-cap moves early in **A_2_C**→**A_2_O** gating.

**Figure 4:**
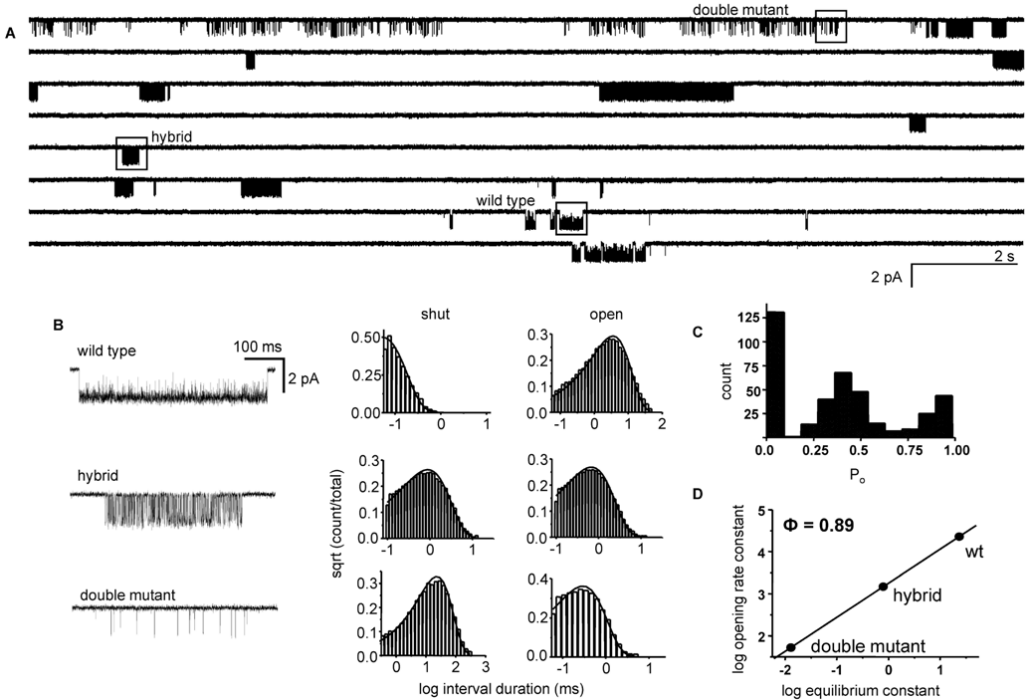
Analysis of αP265K hybrid AChRs. Hybrids are AChRs in which only one of the two α-subunits has been mutated. (A) Low time-resolution view of a continuous current trace showing wild-type, hybrid, and double mutant clusters activated by 500 µM ACh. (B) Expanded view of clusters boxed in A, plus interval duration histograms. (C) Cluster open probability (P_o_) for the patch shown in panel A. The clusters with the highest P_o_ correspond to wild-type receptors, those of the intermediate population correspond to hybrid receptors, and those with the lowest P_o_ are doubly-mutated AChRs. The total number of clusters was 402. (D) REFER analysis shows that the fold-change in K_eq_ for the hybrid is approximately equal to the square root of the fold change for the double mutant, thus the effect of each mutation with regard to A_2_C vs. A_2_O energy changes is equal and independent. The slope of the REFER (Φ) is similar for single- and double-mutant constructs, suggesting that the gating motions of P265 in each α-subunit are approximately synchronous.

There are two α-subunits per AChR. To address the possibility that an M2 mutation in each subunit might contribute unequally to the fold change in K_eq_ or moves at a different point in the gating reaction as does its partner, we expressed hybrid AChRs having one mutated and one wt α subunit (Fig. 4 and Methods). In cells that were transfected with both wt and αP265K subunit cDNAs (along with wt β, δ, and ε), three kinetically distinct populations of clusters were apparent. One had a K_eq_ similar to wt AChRs (38), one had a K_eq_ similar to the αP265K double mutant (0.015), and the remaining group had a K_eq_ that was intermediate (0.76). We attribute this intermediate population to hybrid AChRs that contain one wt and one mutated α subunit. This pattern, a single hybrid class with a fold-change in K_eq_ (50.3) that is approximately equal to the square root of the fold-change of the double mutant (2542), indicates that each αP265K mutation makes an approximately equal and energetically-independent contribution to K_eq_. Further, the Φ value for the αP265K hybrid was similar to that of the double mutant (Fig. 4D), which suggests that at this position the two α subunits move approximately synchronously in the reaction.

**Figure 5:**
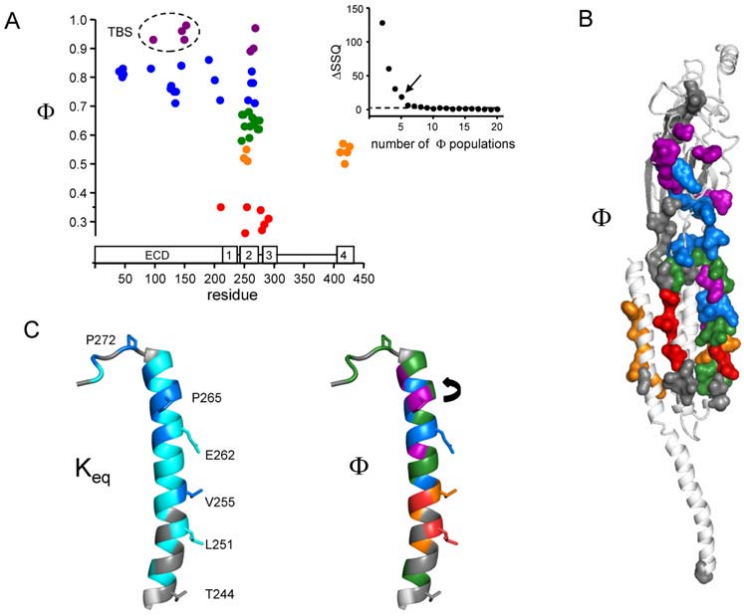
K_eq_ and Φ of the α subunit. (A) Population analysis of Φ in the α subunit. Φ-values of 55 different residues plotted as a function of sequence position (≥2 mutants and >5-fold range in K_eq_). Subunit domains are shown along the x-axis. Each residue was assigned to a Φ population by using a statistical algorithm (see below and Methods). The population means are: purple, 0.94; blue, 0.78; green, 0.64; orange, 0.54 and red, 0.31. Φ-values (Table 2) may reflect the relative timing of gating movements: purple/blue is early, green is intermediate and orange/red is late. High-Φ residues in the TBS are circled. *Inset,* The number of Φ populations (n) was estimated from the sum-squares deviation (SSQ). SSQ decreases significantly as n is increased from n = 2–5, but decreases more slowly between n = 6–20. The most likely number of Φ populations is 5. (B) Map of Φ in the α subunit. Residues are colored according to Φ value (see panel A for color code). The TBS and M2-cap (purple) move at the outset, and the equatorial residues (red) move near the end, of the channel-opening process. (C) Functional maps of αM2 and αM2-M3 linker (α244–α276). M2 residues T244, L251 and E262 face the lumen of the pore. *Left,* Residues colored according to the range for the fold-change in K_eq_: >1000-fold (blue), 10–1000 fold (cyan) and <10-fold (grey) (Table 2). αM2-cap residues experience large energy differences (‘move’) between C and O, whereas many mutants of residues near the cytoplasmic limit of the channel are iso-energetic, which may indicate relatively smaller structural changes. The three biggest excursions in K_eq_ were observed for αP272, αP265 and αV255. *Right*, residues colored according to Φ value (see panel A for color code). Most of the residues in the αM2-cap move ‘early’ in gating (purple and blue), before those in the M2-M3 linker and much of M2 (green). Three cap residues (αI260, αP265 and αS268) have the same Φ value as those for residues at the transmitter binding sites (see panel A). In αM2, residues near the equator have the lowest Φ values and, therefore, move last in C→O gating. *Arrow,* we speculate that when the channel opens, αP265 rotates to position its side chain in the lumen of the channel.

Population analyses of α subunit Φ-values are shown in Fig. 5. Considering all 55 residues for which Φ has been measured, there are most likely five Φ populations, with mean (s.e.m.) values of 0.94 (0.03), 0.78 (0.05), 0.64 (0.03), 0.54 (0.02), and 0.31 (0.04). In the αM2-cap, three residues [αI260 (18′), αP265 (23′) and αS268 (26′)] belong to the highest, four [αV261 (19′), αE262 (20′), αI264 (22′) and αT267(25′)] to the next-highest and the rest [αL263 (21′), αS266 (24′), αS269 (27′) and αA270 (28′)] to the middle Φ-population. αM2-cap residues exhibit higher Φ values than their flanking segments. αI260, αP265 and αS268 have Φ values that are similar to those for amino acids located at the transmitter binding sites (Fig. 5A) [20], [21], [22].

The single-site association and dissociation rate constants (k_+_ and k_−_) and equilibrium dissociation constant (k_+_/k_−_ = K_d_) for ACh binding to the closed conformation were determined for one mutant construct, αE262L (Fig. 2). In this mutant, K_d_ = 155 µM, which is similar to measurements for wild-type AChRs exposed to 140 mM NaCl (100–150 µM [20], [23]). The association and dissociation rate constants in the mutant, k_+_ = 102 µM^−1^s^−1^ and k_−_ = 15,873 s^−1^, were also not greatly different from the wt values (k_+_ = 167 µM^−1^s^−1^ and k_-_ = 24,745 s^−1^; [21]). The failure of this mutation to change K_d_ agrees with similar measurements for three other αM2-cap mutants, αT267I and A, and αS268I [17].

The substitution of a Q at position αE262 (the charged ring) was previously shown to reduce the single-channel conductance by ∼50% [14]. For all constructs, we estimated both the single-channel current amplitude in the absence of channel block (measured at a low agonist concentration) as well as the equilibrium constant for channel block by the agonist (K_B_) (Table S2). Excluding lysine substitutions, the average effect of the mutations on the single-channel current amplitude was substantial for only two positions, αI264 (22′) and αP265 (23′). At four positions the effects were moderate [αE262 (20′), αS266 (24′), αT267 (25′) and αS268 (26′)], while at three the effects were insignificant [αI260 (18′), αV261 (19′), and αL263 (21′)]. Positively-charged side chains were substituted at four positions and caused a large decrease (by ∼75%) in the current at αE262 (K and R) and αP265 (K), had a moderate effect at αL263 (K) and had no effect at αS266 (K). Note that the average consequence of a charge-removal mutation (A, C, F, G, L or V) at αE262 (in both α subunits) was a modest 32% reduction in the current amplitude.

Agonist molecules can bind to the pore and block ionic conduction. In our experimental conditions, the equilibrium dissociation constant for this blockade (K_B_) in wt AChRs is ∼1.9 mM for ACh [8] and ∼13 mM for choline [24]. We estimated the effects of mutations on K_B_ at 5 different cap residues (see Methods and Table S2). Only three mutations had a significant effect: αE262T (9-fold increase for ACh), αI264L (16-fold decrease for choline) and αP265T (5.8-fold decrease for choline). These results suggest that the side chains of the αM2 cap domain do not have a strong effect on equilibrium block by agonist molecules.

Occupancy of the cap domain by certain ligands stabilizes desensitized AChRs. For all constructs, we estimated an apparent rate for entry into long-lived desensitized states, k^*^_+D_ (Table S3). Surprisingly, most of the mutations had little, if any, effect on this rate. The biggest effects on k^*^_+D_ were in αI264L and αS266K (∼10-fold increase) and αL263E (∼2-fold decrease). Although the rate of recovery from desensitization and the number of channels in the patch both contribute to the overall frequency of clusters, we observed no striking change in this parameter for the mutants. Overall, the effects of αM2-cap mutations on desensitization are quite modest, especially when compared to their substantial effects on gating. This result suggests that NCIs increase equilibrium desensitization mainly by perturbing regions of the AChR other than the αM2-cap, and that point side chain substitutions in this region do not mimic these perturbations. We hypothesize that the previously-reported effects of cap mutations on the macroscopic desensitization rate [19], [25] arise from their effects on K_eq_ rather than on microscopic desensitization rate constants.

Overall, αM2-cap mutations have substantial effects on gating but comparatively small effects on agonist binding, channel conductance, channel block and desensitization. The insertion of a positively charge side chain at αE262 (20′) and αP265 (23′) significantly reduces the single-channel current amplitude, which is consistent with the notion that these residues face the open pore and that there is a charged ring in this domain that influences ionic conductance.

## Discussion

The residues of the pore-lining αM2 helix, along with the M2 segments from non-α subunits, form several important functional elements. These include NCI binding sites, a charged ring, residues in the pore that control conductance and an ion selectivity filter (Fig. 5C). All 27 αM2 residues (αT244-αA270) have been examined with respect to the effects of mutations on K_eq_ and Φ (Table 2). We cannot, from our experiments and the available AChR structures, correlate the magnitude of the observed changes in K_eq_ with the magnitudes of the gating motions. However, the large excursions in K_eq_ caused by side chain substitutions at most positions show that most of αM2 changes its structure, dynamics or both between **A_2_C** and **A_2_O**. Residues of the αM2-cap show particularly large excursions in K_eq_ while those in the cytoplasmic portion of αM2 show relatively smaller changes (Fig. 5B). This pattern supports the notion that the most significant **C**↔**O** conformational changes in αM2 (and δM2 [26]) occur at and above the equator [5].

αM2-cap Φ values are higher than for the rest of αM2, which is consistent with the “conformational wave” framework for AChR gating insofar as this domain is near the extracellular limit of the helix and moves prior to the (low-Φ) equatorial zone in channel-opening. The pattern of Φ in the αM2-cap is, however, surprising in two respects. First, αM2-cap Φ values are higher than those of residues in the M2-M3 linker, cys-loop and loop 2, all of which are located between the cap and the TBS. Three αM2-cap residues [αI260 (18′), αP265 (23′) and αS268 (26′)] have Φ-values that cannot be distinguished from those of TBS residues. If Φ reflects the relative timing of gating motions, this result indicates that the gating movements in these two apparently-unconnected regions are approximately synchronous and occur at the outset of the channel-opening process. Second, the map of the entire αM2 segment is complex, with all five Φ-values represented (Fig. 5B). With the temporal interpretation, this suggests that the gating movements in this helix are highly asynchronous, whereas we might expect that side chain motions of such a secondary structural element would either be synchronous, or, perhaps, constitute a continuous, top-to-bottom sequential conformational cascade.

Although we cannot resolve these two conundrums, we can offer some possible explanations.

1. *Unknown linkage elements*. There is no obvious structural connection between the TBS and the αM2-cap in the *Torpedo* AChR structure, where the tip of loop A (residue αD97 [20]; Φ = 0.93±0.02) and cap residue αS268 (Φ = 0.97±0.11) are separated by ∼17 Å. It is difficult to imagine that agonist-triggered gating structural changes at the TBS could propagate, by direct steric interactions, to the αM2-cap. It is possible that the TBS and the αM2-cap are directly linked by high Φ amino acids that have yet to be probed, or that there is a physical connection between these two domains that is invisible in electron density maps (e.g., is electrostatic or arises from the water). For example, gating motions of the αM1 segment, or perturbation of the aqueous milieu consequent to TBS binding or gating motions, might serve to generate the high Φ-values in the αM2-cap.
2. *Incomplete structural information*. Protein movement consequent to agonist binding may move the two high-Φ domains (loop A and the αM2-cap) closer than they are in the unliganded-closed *Torpedo* AChR structural model. This highlights our lack of high resolution structural information regarding the ground states of the **A_2_C**↔**A_2_O** reaction.
3. *Independent gating motions*. Perhaps the motions at the TBS and the cap are completely independent, and these two regions just happen to move early and approximately at the same relative time in the gating reaction in the absence of any direct interactions to couple these motions. This would mean that the microscopic structural transitions that separate **C** and **O** are not strictly sequential. There are precedents for such apparently independent-but-synchronous gating movements. Large distances separate the two α subunits. For example, in both the loop A and M4, residues on the two α subunits are separated by ∼26 Å (αD97) and ∼58 Å (αC418), respectively. Nonetheless, hybrid constructs of these amino acids have approximately the same Φ value [20], [27], as do those of αP265 in the αM2-cap (∼24 Å). Given the complexity of the AChR conformational change, it is not unreasonable to think that separate domains can move independently but approximately at the same time, and will thus have similar experimental Φ values. The αM2 cap and the agonist-occupied TBS may be inherently unstable structures that deform early in the **C**→**O** isomerization.
4. *The interpretation of Φ*. Φ may not reflect time in the αM2-cap domain. The central assumption of the temporal interpretation of Φ is that mutations alter the **C→O** rate constant by changing the transmission coefficient, but the magnitude of k_o_ also reflects transition state (**TS**) energy and, perhaps, heterogeneity. Further, the weights given to these various factors (with regard to k_o_) could be different for different regions of a protein or even for different individual residues. Another assumption of the temporal interpretation is that a side chain undergoes only a single, instantaneous, all-or-none gating movement. It is, however, possible that some side chain atoms (we do not mutate the backbone) are jostled more than once within the reaction, in which case the apparent Φ value will be a weighted average of the relative times and energy changes of such multiple motions. We can imagine that the transition region energy changes of the three cap high-Φ residues (α260, α265 and α268; Φ = 0.92) occur mainly early in the reaction, those in the M2-M3 linker and in much of M2 occur mainly near the middle of the reaction (Φ = 0.64), and that the ‘intermediate’ residues of the cap (α261, α262, α264 and α267; Φ = 0.77) move twice, along with each of these other groups. The possibility of multiple side chain motions is physically plausible but further complicates the interpretation of Φ values.

The resolution of the electron density map of the αM2 cap in the *Torpedo* AChR is not sufficiently high to assess the potential for, or chemical nature of, the specific structural changes in this domain that accompany **C↔O** gating. Also, there are as yet no published structures of a ligand-occupied intact AChR, although there are structural differences between occupied and vacant AChBP [28], [29], [30] and the ECDs of α vs. non-α AChR subunits that may reflect **C** vs. **O** conformations, respectively [31]. In the absence of high resolution structures of the wt and mutant AChRs it is difficult to infer specific structural events based on the functional effects of mutations.

The basic features of the αM2 cap are as follows. It is a ∼9-residue (260–268, which subtends the high-Φ amino acids), segment that is at the C-terminus of a long α-helix. Some cap side chains face the water-accessible, ion permeation pathway while others are close to M1 and M3. There is a conserved Pro near the middle of the segment. In 2bg9.pdb, the modeled Φ/Ψ backbone bonds for αP265 and αI264 are ∼89°/30° and ∼84°/12°, which are outside the typical values for proline (55°/50°) [32] and pre-proline (60°/45°) [32], [33] residues.

We speculate that the central proline (αP265) of the αM2-cap distorts and destabilizes the C-terminal portion of M2, which enables the cap to readily switch its secondary structure during the **C**↔**O** conformational change. This hypothesis accounts for the observations that most cap residues experience large energy changes in gating, and that some appear to move at the outset of channel-opening. The change in the backbone cannot be a full, cis-trans isomerization, because many different side chain substitutions at αP265 support efficient gating. The fact that the effect of a K substitution on the single-channel current amplitude was similar at αE262 (20′) and αP265 (23′) (Table S2) suggests that these two residues are aligned along the pore axis when the AChR is in an open-channel conformation (Fig. 5C). Although the specific structural changes are not revealed in our experiments, we hypothesize that the backbone angles of the central proline and preceding isoleucine change in **C**↔**O** gating, and that this switch in the secondary structure of the αM2-cap permits the translation of ECD motions into the rest of M2 and, thence, to other M2 residues that regulate ionic conductance, including the late-moving 9′ and 12′ residues [5]. This is similar to the suggestion that channel-opening involves an outward tilt of the M2-cap [3], although our experiments suggest this motion may involve a twist. Interestingly, a different experimental approach indicates that there are only minor movements in the M2 helix of the δ subunit in C↔O gating [34].

We now describe a sequence of events in the α subunit channel-opening cascade, based on Φ values and the assumption that mutations mainly affect the transmission coefficient of k_o_. In the following framework, all of the gating motions are stochastic (are characterized by back-and-forth, Brownian dynamics). Also, the reverse sequence describes channel-closing.

- i) Conformational changes consequent to agonist binding destabilize at least two domains of each α subunit, the TBS (loops A, B and C) and the αM2-cap. Residue αK145 in the outer β sheet of the ECD is also destabilized [22]. The gating motions of the TBS residues increase the affinity for ACh by a factor of ∼10,000 [22], [35], but the conductance of the channel remains low. The motion of the TBS announces the exit from the **C** structural ensemble and entry into the **TS** ensemble. The trigger for the change in structure at the TBS is the presence of the agonist itself, but that for the cap region remains obscure.
- ii) The motions of the TBS and αM2-cap trigger those in adjacent domains, including loop 2, the cys-loop and residue αY127 in the inner β sheet of the ECD. These motions are then followed by the movement of residues in the M2-M3 linker and in M2, both within the αM2-cap and below, to the equator and beyond. These intermediate events reflect structural changes that occur within the **TS** ensemble of the reaction, where the TBS affinity remains high but the channel conductance is still low.
- iii) The above gating motions in αM2 destabilize residues αL251(9′) and αT254(12′). It is possible that the movement of these residues serves to change ionic conductance (they act as a ‘gate’), but it is also possible that ions begin to cross the channel rapidly when the protein is still in the short-lived **TS** ensemble (they act as a ‘latch’). At this point in opening the TBS still has a high affinity for agonists, and the movement of the αM2 equator reflects entry into the **O** structural ensemble.

To confirm and complete this gating scenario we will need high resolution structures of intact AChRs in both **A_2_C** and **A_2_O** conformations, more extensive estimates of the energy changes in αM1 and the M2 segments of the non-α subunits, and more sophisticated theories for, and analyses of, the transition state of the gating reaction.

## Methods

Detailed methods are given in Jha et al, (2007) [18]. Briefly, mutant AChRs (64 different mutants of 9 different amino acid positions) were transiently expressed in HEK cells, and single channel currents were recorded in the cell-attached patch configuration at 23°C. The bath and pipette solutions were Dulbecco's phosphate buffered saline containing (in mM): 137 NaCl, 0.9 CaCl_2_, 2.7 KCl, 1.5 KH_2_PO_4_, 0.5 MgCl_2_, and 8.1 Na_2_HPO_4_ (pH 7.3). The currents were filtered at 20 kHz and digitized at a sampling frequency of 50 kHz. Agonist (acetylcholine or choline) was added to the pipette solution. For rate constant measurements, the agonist concentration was approximately five times K_d_ (500 µM ACh or 20 mM choline). Choline was used to activate constructs in which K_eq_ was similar to or larger than in the wt (gain-of-function mutants), and ACh was used to activate constructs in which K_eq_ was smaller than in the wt (loss-of-function mutants). Rate constant estimation (12 kHz bandwidth) was done by using QUB software (www.qub.buffalo.edu). Clusters of individual-channel, diliganded **C**↔**O** activity were usually selected by eye or by using a critical time of 50 ms. Typically, ∼50 clusters were selected in each record. The opening and closing rate constants were estimated from the interval durations by using a maximum likelihood algorithm [36] after imposing a dead time correction of, typically, 25 µs. Φ was estimated as the slope of the rate-equilibrium free energy relationship (REFER), which is a plot of log k_o_ vs. log K_eq_ (Fig. 3). Each point in the REFER represents the mean of at least three different patches for a single mutant construct.

We could not determine the gating rate constants for αP265F and αP265L because no currents were detected (8 patches each, 10 min/patch). Also, rate constructs could not be measured for the constructs αI260F, αS266L, αS266Y and αT267F because the openings were not organized into well-defined clusters at 500 µM ACh, most likely because these constructs had exceeding small values of K_eq_. Clusters from αS266C showed two distinct kinetic patterns, and k_c_ and k_o_ were estimated separately for each. αS268Y showed multiple kinetics patterns so no rate constants were estimated for this mutant. In total, rate constants were estimated for 57 of the 64 constructs that were examined (Table S1).

The K_d_ for acetylcholine was estimated only for the αE262L mutant (Fig. 2). Open and closed interval durations were obtained at three different ACh concentrations (30, 50 and 100 µM). The two agonist binding sites were assumed to be equivalent and independent [37] and the interval durations at all three concentrations were fitted together by using a **C**↔**AC**↔**A_2_C**↔**A_2_O** kinetic model (A = agonist) that had four rate constants as free parameters: single-site association (k_+_, scaled by [A]), single-site dissociation (k_−_), k_o_, and k_c_.

In the REFERs (Fig. 3), the wt values used to normalize k_o_ and K_eq_ were 120 s^−1^ and 0.046 for AChRs activated by choline and 48,000 s^−1^ and 28.2 for AChRs activated by ACh. The slope of the REFER was estimated by an unweighted, linear fit in Origin Pro 7.0. All structures were displayed by using PYMOL (DeLano Scientific).

The number of Φ populations (Fig. 5A) was estimated statistically by using a cluster-detection algorithm (SKM), which assumes each population had a Gaussian distribution with an independent mean and s.d [5]. The overall sum-square deviation (SSQ) was estimated assuming n = 2 to 20 populations. 300 random starting assignments were used for each value of n.

In the experiments concerning hybrid AChRs (Fig. 4), cells were transfected with both wild-type and mutant (P265K) α subunit cDNAs in a 1:3 ratio, together with wild-type β, δ, and ε subunit cDNAs. All recordings showed populations of clusters that could be distinguished statistically according to the cluster open probability (P_o_), corresponding to wild-type, hybrid (containing one wild-type and one mutant α subunit) or double-mutant AChRs. Clusters were either selected by eye or defined using a critical time of 50 ms and were segregated statistically (segmentation k-means algorithm; SKM) into separate populations for subsequent kinetic analyses with only the cluster P_open_ as the discrimination criterion. Clusters that had P_o_ values that were >1 SD from the corresponding population mean were rejected from these analyses.

In neuromuscular AChRs desensitization appears to proceed mainly from the **A_2_O** state [8] or from a transition micro-state that is near **A_2_O** [4], [7]. An approximate rate of entry into long-lived desensitized states was determined by computing the inverse of the product of the cluster duration times the cluster open probability: k^*^_+D_ = (τ_c_P_o_)^−1^ (Table S3). This parameter is a rough estimate of the net rate of exiting **A_2_O** into a long-lived **D** state.

An estimate of the equilibrium constant for channel block by the agonist (K_B_) was determined for each construct from the relationship K_B_ = [A]i_B_/(i_0_−i_B_), where [A] is the agonist concentration, i_0_ is the current amplitude in the absence of channel block (30 µM ACh or 200 µM choline), and i_B_ is the current amplitude at high [A] (Table S2). For normalization, the wt parameters were K_B_ = 1.9 mM for ACh [8] and 13 mM for choline [24]. The fractional reduction in amplitude at 500 µM ACh was small (∼20% in the wt), and, because of errors in the estimate of the membrane voltage, the K_B_ estimates for such ACh-activated currents were imprecise. Therefore, only mutants that showed a >50% decrease in current amplitude at 500 µM ACh were used for K_B_ estimation. For choline-activated constructs, the fractional reduction in the wt current amplitude at 20 mM is more substantial (∼60%) so K_B_ could be estimated for all.

## Supporting Information

Table S1 Rate and equilibrium constant estimates for the αM2-cap Mutants (260–268)
(0.16 MB DOC)

Table S2 Conductance and Channel Block for αM2-cap Mutants (260–268)
(0.14 MB DOC)

Table S3 Apparent Desensitization Rates for αM2-cap Mutants (260–268)
(0.12 MB DOC)

Figure S1 Single-channel current traces of various αM2 cap mutants
(0.82 MB TIF)
