# Supplementary material for: Gating at the Mouth of the Acetylcholine Receptor Channel: Energetic Consequences of Mutations in the αM2-Cap
Source: PLoS One. 2008 Jun 25;3(6):e2515. doi: 10.1371/journal.pone.0002515 (PMC2429975; doi:10.1371/journal.pone.0002515)
Supplement: Table S1 — Rate and equilibrium constant estimates for the αM2-cap Mutants (260–268) (0.16 MB DOC) [file pone.0002515.s001.doc]

**TABLE S1: Rate and equilibrium constant estimates for the αM2-cap mutants (260-268)**

| Construct | k_o_ (s^-1^) | k_c_^obs^ (s^-1^) | k_c_^cor^ (s^-1^) | K_eq_ (k_o_/ k_c_^cor^) | Agonist | Fold-change  in K_eq_ (mut/wt) | *n* |
| --- | --- | --- | --- | --- | --- | --- | --- |
| wt^a^ | 120 | - | 2583 | 0.046 | Cho | 1 |  |
| wt^b^ | 48000 | - | 1700 | 28.2 | ACh | 1 |  |
|  |  |  |  |  |  |  |  |
| I260A | 251 (27) | 3031 (411) | 2765 (84) | 0.09 (0.01) | ACh | 0.003 | 3 |
| I260G | 708 (149) | 2033(70) | 4000 (169) | 0.18 (0.04) | ACh | 0.006 | 4 |
| I260M | 16610 (636) | 4162 (626) | 2425 (69) | 6.86 (0.46) | ACh | 0.243 | 2 |
| I260S | 1502 (141) | 2457 (41) | 2439 (159) | 0.61 (0.02) | ACh | 0.022 | 4 |
| I260W | 524 (125) | 4176 (406) | 4063 (220) | 0.13 (0.03) | ACh | 0.005 | 4 |
|  |  |  |  |  |  |  |  |
| V261A | 5987 (733) | 704 (42) | 758 (30) | 7.91 (0.89) | ACh | 0.280 | 5 |
| V261D | 204 (63) | 6243 (387) | 7805 (795) | 0.03 (0.01) | ACh | 0.001 | 3 |
| V261E | 744 (59) | 753 (200) | 824 (52) | 0.91 (0.07) | ACh | 0.032 | 3 |
| V261F | 1307 (133) | 1344 (148) | 2579 (135) | 0.50 (0.02) | ACh | 0.018 | 3 |
| V261S | 10610 (550) | 849 (83) | 1202 (97) | 9.01 (0.93) | ACh | 0.320 | 4 |
| V261T | 18130 (842) | 1717 (159) | 2599 (16) | 6.98 (0.37) | ACh | 0.248 | 2 |
|  |  |  |  |  |  |  |  |
| E262A | 16580 (967) | 609 (97) | 1755 (51) | 9.45 (0.43) | ACh | 0.335 | 3 |
| E262C | 45.5 (1.5) | 2751 (85) | 3966 (6.45) | 0.01 (0.001) | Cho | 0.217 | 2 |
| E262D | 377 (60) | 1582 (57) | 10050 (783) | 0.04 (0.007) | Cho | 0.870 | 3 |
| E262F | 9031 (1123) | 2049 (109) | 2974 (47) | 3.05 (0.42) | ACh | 0.108 | 3 |
| E262G | 905 (44) | 649 (51) | 996 (32) | 0.92 (0.07) | Cho | 20.00 | 5 |
| E262L | 2760 (261) | 1008 (132) | 1099 (38) | 4.66 (0.60) | ACh | 0.165 | 3 |
| E262V | 22010 (711) | 2889 (176) | 1301 (38) | 16.92 (0.25) | ACh | 0.60 | 3 |
| E262T | 24320 (2904) | 1277 (228) | 1650 (105) | 14.91 (2.71) | ACh | 0.529 | 2 |
| E262K | 12870 (1020) | 5982 (117) | 548 (1.45) | 23.5 (1.92) | ACh | 0.833 | 3 |
|  |  |  |  |  |  |  |  |
| L263A | 485 (81) | 616 (60) | 5325 (226) | 0.09 (0.02) | Cho | 1.957 | 3 |
| L263C | 366 (8.96) | 682 (26) | 5553 (30) | 0.07 (0.002) | Cho | 1.522 | 3 |
| L263D | 1153 (97) | 1392 (266) | 4679 (45) | 0.25 (0.02) | Cho | 5.435 | 2 |
| L263E | 1367 (137) | 280 (17) | 2418 (48) | 0.56 (0.05) | Cho | 12.174 | 3 |
| L263F | 772 (106) | 888 (91) | 8449 (859) | 0.09 (0.01) | Cho | 1.957 | 3 |
| L263G | 1461 (91) | 810 (78) | 849 (33) | 1.73 (0.17) | Cho | 37.609 | 3 |
| L263I | 14070 (1212) | 1640 (106) | 1990 (86) | 7.13 (0.88) | ACh | 0.253 | 3 |
| L263K | 475 (67) | 1004 (40) | 1287 (9.29) | 0.37 (0.05) | Cho | 8.043 | 3 |
| L263Y | 2279 (296) | 374 (20) | 786 (8.04) | 2.91 (0.41) | Cho | 63.261 | 3 |
|  |  |  |  |  |  |  |  |
| I264A | 3796 (460) | 14230 (1390) | 18570 (958) | 0.21 (0.03) | ACh | 0.007 | 4 |
| I264E | 5454 (574) | 2735 (656) | 2155 (975) | 8.54 (6.64) | ACh | 0.303 | 3 |
| I264F | 1599 (101) | 4996 (457) | 18240 (719) | 0.09 (0.004) | Cho | 1.957 | 3 |
| I264G | 279 (79) | 5477 (922) | 17820 (2325) | 0.02 (0.002) | Cho | 0.435 | 3 |
| I264L | 2403 (130) | 1451 (205) | 2966 (184) | 0.82 (0.08) | Cho | 17.826 | 3 |
| I264M | 10444 (2293) | 2702 (107) | 2161 (51) | 6.77 (1.07) | ACh | 0.240 | 3 |
| I264S | 6134 (313) | 8778 (779) | 12710 (915) | 0.49 (0.02) | ACh | 0.017 | 4 |
|  |  |  |  |  |  |  |  |
| P265A | 13920 (1008) | 1108 (51) | 1445 (104) | 10.21 (1.19) | ACh | 0.36 | 4 |
| P265G | 3503 (849) | 6689 (150) | 8361 (187) | 0.42 (0.09) | ACh | 0.02 | 2 |
| P265K | 93 (10) | 3320 (88) | 4150 (111) | 0.022 (0.002) | ACh | 0.0008 | 3 |
| P265S | 8022 | 2156 | 2695 | 2.98 | ACh | 0.11 | 1 |
| P265T | 1296 (162) | 1437 (299) | 3837 (799) | 0.377 (0.11) | Cho | 8.20 | 3 |
| S266A | 4362 (566) | 1767 (165) | 1063 (51) | 4.16 (0.73) | ACh | 0.148 | 3 |
| S266C_(low Po)_ | 320 (38) | 6824 (1321) | 3438 (296) | 0.10 (0.02) | ACh | 0.004 | 3 |
| S266C_(mid Po)_ | 1049 (237) | 4179 (619) | 3290 (249) | 0.32 (0.06) | ACh | 0.011 | 3 |
| S266D | 2144 (274) | 894 (43) | 646 (13) | 3.34 (0.48) | ACh | 0.118 | 3 |
| S266E | 1082 (250) | 6342 (598) | 7669 (145) | 0.14 (0.03) | ACh | 0.005 | 4 |
| S266K | 630 (96) | 11700 (1347) | 15500 (562) | 0.04 (0.006) | ACh | 0.001 | 3 |
| S266T | 6313 (394) | 3914 (178) | 6263 (246) | 1.01 (0.03) | ACh | 0.036 | 3 |
|  |  |  |  |  |  |  |  |
| T267A | 2169 (431) | 3838 (189) | 5262 (601) | 0.77 (0.24) | ACh | 0.027 | 5 |
| T267D | 4858 (465) | 3990 (205) | 8463 (522) | 0.59 (0.09) | ACh | 0.021 | 4 |
| T267V | 1244 (153) | 5653 (1589) | 6017 (779) | 0.22 (0.05) | ACh | 0.008 | 3 |
|  |  |  |  |  |  |  |  |
| S268A | 1054 (94) | 1137 (104) | 11590 (199) | 0.09 (0.008) | Cho | 1.957 | 3 |
| S268E | 10820 (912) | 767 (103) | 539 (12) | 5.38 (0.42) | Cho | 116.957 | 3 |
| S268D | 3604 (469) | 2864 (218) | 4780 (191) | 0.76 (0.13) | Cho | 16.522 | 3 |
| S268F | 2298 (252) | 771 (99) | 1064 (126) | 2.19 (0.24) | ACh | 0.078 | 4 |
| S268L | 2403 (112) | 1875 (218) | 4426 (8.98) | 0.69 (0.02) | ACh | 0.024 | 2 |
| S268T | 174 (20) | 850 (99) | 4410 (115) | 0.04 (0.005) | Cho | 0.870 | 4 |

k_o_, apparent opening rate constant; k_c_^obs^, apparent closing rate constant; k_c_^cor^, closing rate constant after correction for channel block; *n*, number of patches. Values are mean (±s.e.m.) ^a^From [38]; ^b^From [18].
