# Supplementary material for: Gating at the Mouth of the Acetylcholine Receptor Channel: Energetic Consequences of Mutations in the αM2-Cap
Source: PLoS One. 2008 Jun 25;3(6):e2515. doi: 10.1371/journal.pone.0002515 (PMC2429975; doi:10.1371/journal.pone.0002515)
Supplement: Table S2 — Conductance and Channel Block for αM2-cap Mutants (260–268) (0.14 MB DOC) [file pone.0002515.s002.doc]

**TABLE S2: Conductance and Channel Block for αM2-cap Mutants (260-268)**

| **Mutant** | **Agonist** | **i_0_ (pA)** | **i_B_ (pA)** | **K_B_ (mM)** | **i_0_/7 (pA)** | **K_B_(mut)/K_B_(wt)** |
| --- | --- | --- | --- | --- | --- | --- |
| I260A | ACh | 5.8 | 5.2 (0.16) | - | 0.83 | - |
| I260G | ACh | 5.6 | 4.2 (0.17) | - | 0.80 | - |
| I260M | ACh | 7.7 | 6.1 (0.18) | - | 1.10 | - |
| I260S | ACh | 5.3 | 5.0 (0.32) | - | 0.75 | - |
| I260W | ACh | 7.8 | 5.5 (0.28) | - | 1.12 | - |
|  |  |  |  |  |  |  |
| V261A | ACh | 6.7 | 5.7 (0.24) | - | 0.95 | - |
| V261D | ACh | 4.7 | 4.4 (0.41) | - | 0.67 | - |
| V261E | ACh | 7.6 | 5.1 (0.31) | - | 1.09 | - |
| V261F | ACh | 7.1 | 4.9 (0.26) | - | 1.01 | - |
| V261S | ACh | 8.1 | 5.0 (0.41) | - | 1.16 | - |
| V261T | ACh | 8.0 | 6.3 (0.04) | - | 1.14 | - |
|  |  |  |  |  |  |  |
| E262A | ACh | 5.3 | 3.5 (0.10) | - | 0.76 | - |
| E262C | Cho | 3.8 | 2.1 (0.003) | 26.3 (0.09) | 0.54 | 2.02 (0.01) |
| E262D | Cho | 5.1 | 2.1 (0.16) | 13.7 (1.78) | 0.73 | 1.06 (0.14) |
| E262F | ACh | 6.6 | 4.4 (0.07) | - | 0.94 | - |
| E262G | Cho | 4.3 | 1.7 (0.05) | 12.6 (0.65) | 0.62 | 0.97 (0.05) |
| E262L | ACh | 4.3 | 4.4 (0.15) | - | 0.61 | - |
| E262V | ACh | 4.6 | 4.8 (0.14) | - | 0.65 | - |
| E262T | ACh | 6.7 | 1.7 (0.11) | 0.2 (0.03) | 0.96 | 0.09 (0.01) |
| E262K | ACh | 1.9 | 1.7 (0.01) | - | 0.27 | - |
|  |  |  |  |  |  |  |
| L263A | Cho | 4.9 | 1.8 (0.08) | 11.2 (0.74) | 0.70 | 0.86 (0.06) |
| L263C | Cho | 5.5 | 1.6 (0.01) | 8.4 (0.06) | 0.79 | 0.65 (0.01) |
| L263D | Cho | 5.8 | 1.6 (0.02) | 7.4 (0.1) | 0.83 | 0.57 (0.01) |
| L263E | Cho | 6.1 | 1.8 (0.04) | 8.5 (0.25) | 0.88 | 0.65 (0.02) |
| L263F | Cho | 6.8 | 1.7 (0.18) | 6.5 (0.96) | 0.98 | 0.50 (0.07) |
| L263G | Cho | 4.4 | 3.2 (0.12) | 53.8 (6.9) | 0.63 | 4.14 (0.53) |
| L263I | ACh | 7.0 | 5.1 (0.22) | - | 1.00 | - |
| L263K | Cho | 4.1 | 1.6 (0.01) | 13.3 (0.15) | 0.58 | 1.02 (0.01) |
| L263Y | Cho | 6.9 | 2.1 (0.04) | 8.9 (0.13) | 0.99 | 0.69 (0.01) |
|  |  |  |  |  |  |  |
| I264A | ACh | 4.1 | 2.9 (0.14) | - | 0.59 | - |
| I264E | ACh | 6.4 | 4.1 (0.16) | - | 0.92 | - |
| I264F | Cho | 2.5 | 1.7 (0.1) | 43 (3.95) | 0.36 | 3.31 (0.30) |
| I264G | Cho | 4.2 | 1.6 (0.21) | 12.4 (2.65) | 0.60 | 0.96 (0.20) |
| I264L | Cho | 2.9 | 2.6 (0.16) | 210.4 (36.2) | 0.41 | 16.18 (4.32) |
| I264M | ACh | 4.2 | 2.6 (0.06) | - | 0.60 | - |
| I264S | ACh | 3.8 | 3.6 (0.23) | - | 0.54 | - |
|  |  |  |  |  |  |  |
| P265A | ACh | 2.5 | 2.1 (0.03) | -- | 0.36 | - |
| P265G | ACh | 4.7 | 4.7 (0.39) | - | 0.67 | - |
| P265K | ACh | 2.06 | 1.88 (0.11) | - | 0.29 | - |
| P265S | ACh | 5.0 | 5.02 (0.31) | - | 0.71 | - |
| P265T | Cho | 1.81 | 1.43 (0.01) | 75.26 (2.90) | 0.26 | 5.79 (0.22) |
|  |  |  |  |  |  |  |
| S266A | ACh | 4.43 | 4.09 (0.20) | - | 0.63 | - |
| S266C_(low Po)_ | ACh | 4.48 | 4.96 (0.40) | - | 0.64 | - |
| S266C_(mid Po)_ | ACh | 4.48 | 5.17 (0.37) | - | 0.64 | - |
| S266D | ACh | 5.64 | 4.55 (0.09) | - | 0.81 | - |
| S266E | ACh | 6.58 | 4.81 (0.09) | - | 0.94 | - |
| S266K | ACh | 7.0 | 4.60 (0.17) | - | 1.00 | - |
| S266T | ACh | 4.63 | 4.11 (0.16) | - | 0.66 | - |
|  |  |  |  |  |  |  |
| T267A | ACh | 4.25 | 4.9 (0.46) | - | 0.61 | - |
| T267D | ACh | 6.06 | 4.32 (0.27) | - | 0.87 | - |
| T267V | ACh | 4.25 | 4.1 (0.47) | - | 0.61 | - |
|  |  |  |  |  |  |  |
| S268A | Cho | 4.18 | 1.57 (0.03) | 12.03 (2.35) | 0.60 | 0.93 (0.08) |
| S268D | Cho | 4.92 | 1.51 (0.06) | 8.86 (1.12) | 0.70 | 0.68 (0.05) |
| S268E | Cho | 7.98 | 1.57 (0.04) | 4.90 (0.75) | 1.14 | 0.38 (0.07) |
| S268F | ACh | 4.23 | 4.90 (0.47) | - | 0.60 | - |
| S268L | ACh | 6.56 | 4.93 (0.01) | - | 0.94 | - |
| S268T | Cho | 4.9 | 1.75 (0.05) | 11.11 (0.12) | 0.7 | 0.85 (0.03) |

i_0_, current amplitude at 30 μM ACh or 200 µM choline (7 pA in the wt); i_B_, current amplitude at 500 µM ACh or 20 mM choline; K_B_, equilibrium dissociation constant for channel block by the agonist; i_0_/7, normalized current amplitude; K_B_(mut)/K_B_(wt), normalized K_B_.
