# Supplementary material for: Gating at the Mouth of the Acetylcholine Receptor Channel: Energetic Consequences of Mutations in the αM2-Cap
Source: PLoS One. 2008 Jun 25;3(6):e2515. doi: 10.1371/journal.pone.0002515 (PMC2429975; doi:10.1371/journal.pone.0002515)
Supplement: Table S3 — Apparent Desensitization Rates for αM2-cap Mutants (260–268) (0.12 MB DOC) [file pone.0002515.s003.doc]

**TABLE S3: Apparent Desensitization Rates for αM2-cap Mutants (260-268)**

| **Mutant** | **Agonist** | **τ_c_ (ms)** | **P_o_** | **k_+D_ (mut) s^-1^**  **(1/τ_c_ P_o_)** | **k_+D_(mut)/k_+D_(wt)** |
| --- | --- | --- | --- | --- | --- |
| I260A | ACh | 783 (10) | 0.06 (0.004) | 20.0 (0.2) | 5.68 (0.06) |
| I260G | ACh | 484 (109) | 0.24 (0.04) | 8.6 (1.05) | 2.45 (0.58) |
| I260M | ACh | 303 (91) | 0.78 (0.08) | 4.2 (0.45) | 1.21 (0.13) |
| I260S | ACh | 460 (78) | 0.34 (0.01) | 6.5 (1.66) | 1.84 (0.48) |
| I260W | ACh | 265 (68) | 0.15 (0.05) | 24.5 (4.8) | 6.98 (1.6) |
|  |  |  |  |  |  |
| V261A | ACh | 235 (7.5) | 0.83 (0.02) | 5.1 (0.03) | 1.46 (0.01) |
| V261D | ACh | 2156 (541) | 0.02 (0.01) | 27.3 (4.3) | 7.78 (1.2) |
| V261E | ACh | 698 (66) | 0.56 (0.06) | 2.5 (0.01) | 0.72 (0.07) |
| V261F | ACh | 511 (117) | 0.46 (0.05) | 4.3 (1.28) | 1.21 (0.37) |
| V261S | ACh | 235 (60) | 0.90 (0.04) | 4.7 (1.61) | 1.35 (0.47) |
| V261T | ACh | 338 (31) | 0.88 (0.11) | 3.4 (0.21) | 0.96 (0.06) |
|  |  |  |  |  |  |
| E262A | ACh | 118 (12) | 0.86 (0.04) | 9.9 (0.89) | 2.81 (0.25) |
| E262C | Cho | 2369 (566) | 0.02 (0.001) | 22.2 (3.17) | 6.33 (0.90) |
| E262D | Cho | 1178 (155) | 0.15 (0.01) | 5.8 (0.98) | 1.65 (0.28) |
| E262F | ACh | 296 (23) | 0.69 (0.04) | 4.9 (0.21) | 1.39 (0.06) |
| E262G | Cho | 448 (121) | 0.40 (0.06) | 5.6 (0.21) | 1.59 (0.06) |
| E262L | ACh | 467 (56) | 0.49 (0.07) | 4.3 (0.17) | 1.24 (0.05) |
| E262V | ACh | 341 (55) | 0.86 (0.15) | 3.4 (0.28) | 0.97 (0.08) |
| E262T | ACh | 560 (30) | 0.72 (0.03) | 2.5 (0.28) | 0.71 (0.08) |
| E262K | ACh | 398 (22) | 0.56 (0.05) | 4.6 (0.17) | 1.31 (0.05) |
|  |  |  |  |  |  |
| L263A | Cho | 267 (73) | 0.43 (0.03) | 7.0 (1.20) | 2.54 (0.34) |
| L263C | Cho | 766 (67) | 0.30 (0.01) | 4.4 (0.32) | 1.24 (0.09) |
| L263D | Cho | 1140 (7.5) | 0.31 (0.09) | 2.8 (0.51) | 0.81 (0.15) |
| L263E | Cho | 680 (258) | 0.7 (0.01) | 2.1 (0.44) | 0.60 (0.13) |
| L263F | Cho | 467 (69) | 0.45 (0.03) | 4.8 (1.41) | 1.36 (0.40) |
| L263G | Cho | 178 (41) | 0.59 (0.05) | 9.5 (0.73) | 2.71 (0.21) |
| L263I | ACh | 393 (81) | 0.61 (0.06) | 4.2 (1.6) | 1.19 (0.31) |
| L263K | Cho | 1037 (231) | 0.24 (0.03) | 4.0 (1.22) | 1.15 (0.46) |
| L263Y | Cho | 445 (112) | 0.67 (0.04) | 3.4 (0.62) | 0.96 (0.18) |
|  |  |  |  |  |  |
| I264A | ACh | 736 (118) | 0.15 (0.05) | 13.6 (5.19) | 3.87 (1.32) |
| I264E | ACh | 524 (63) | 0.55 (0.01) | 3.5 (0.28) | 0.99 (0.08) |
| I264F | Cho | 429 (146) | 0.21 (0.003) | 11.1 (2.76) | 3.16 (0.79) |
| I264G | Cho | 1857 (440) | 0.04 (0.01) | 13.5 (3.50) | 3.84 (0.98) |
| I264L | Cho | 62 (25) | 0.62 (0.03) | 37.6 (15) | 10.72 (4.32) |
| I264M | ACh | 225 (5) | 0.82 (0.08) | 5.4 (0.51) | 1.54 (0.15) |
| I264S | ACh | 545 (62) | 0.29 (0.01) | 6.3 (1.14) | 1.80 (0.33) |
| P265A | ACh | 368 (25) | 0.72 (0.06) | 3.8 (0.12) | 1.08 (0.52) |
| P265G | ACh | 313 (22) | 0.34 (0.12) | 9.4 (0.8) | 2.68 (0.18) |
| P265K | ACh | 1832 (225) | 0.04 (0.008) | 13.64 (1.12) | 3.89 (0.77) |
| P265S | ACh | 147 | 0.79 | 8.6 | 2.45 |
| P265T | Cho | 458 (55) | 0.74 (0.05) | 3.0 (0.33) | 0.84 (0.12) |
|  |  |  |  |  |  |
| S266A | ACh | 483 (97) | 0.69 (0.02) | 3.1 (0.56) | 0.89 (0.16) |
| S266C_(low Po)_ | ACh | 1646 (187) | 0.08 (0.02) | 7.6 (1.26) | 2.16 (0.48) |
| S266C_(mid Po)_ | ACh | 988 (230) | 0.24 (0.05) | 4.2 (1.15) | 1.20 (0.18) |
| S266D | ACh | 424 (104) | 0.63 (0.05) | 3.7 (0.57) | 1.07 (0.16) |
| S266E | ACh | 1159 (121) | 0.06 (0.01) | 13.9 (2.99) | 3.97 (0.85) |
| S266K | ACh | 479 (72) | 0.06 (0.01) | 33.7 (7.69) | 9.60 (1.12) |
| S266T | ACh | 189 (25) | 0.65 (0.06) | 8.14 (1.77) | 2.32 (0.84) |
|  |  |  |  |  |  |
| T267A | ACh | 597 (53) | 0.29 (0.08) | 6.2 (1.18) | 1.76 (0.34) |
| T267D | ACh | 381 (55) | 0.49 (0.05) | 5.7 (0.96) | 1.63 (0.27) |
| T267V | ACh | 746 (55) | 0.29 (0.09) | 4.6 (0.17) | 1.32 (0.07) |
|  |  |  |  |  |  |
| S268A | Cho | 1105 (305) | 0.29 (0.12) | 3.1 (0.18) | 0.89 (0.11) |
| S268E | Cho | 183 (2) | 0.65 (0.15) | 8.9 (2.15) | 2.54 (0.61) |
| S268D | Cho | 228 (42) | 0.72 (0.14) | 6.1 (1.22) | 1.74 (0.07) |
| S268F | ACh | 417 (75) | 0.73 (0.18) | 3.3 (0.21) | 0.94 (0.17) |
| S268L | ACh | 503 (44) | 0.48 (0.05) | 4.8 (0.08) | 1.35 (0.07) |
| S268T | Cho | 1482 (321) | 0.097 (0.03) | 7.0 (1.10) | 1.98 (0.41) |

τ_c_, cluster duration; P_o_, cluster open probability; k_+D_, rate of entry into long-lived desensitized states, (τ_c_P_o_)^-1^; k_+D_(mut)/k_+D_(wt), normalized k_+D_ (wt k_+D_ from [8]).
