# Supplementary figures and images for: Gating at the Mouth of the Acetylcholine Receptor Channel: Energetic Consequences of Mutations in the αM2-Cap
Source: PLoS One. 2008 Jun 25;3(6):e2515. doi: 10.1371/journal.pone.0002515 (PMC2429975; doi:10.1371/journal.pone.0002515)

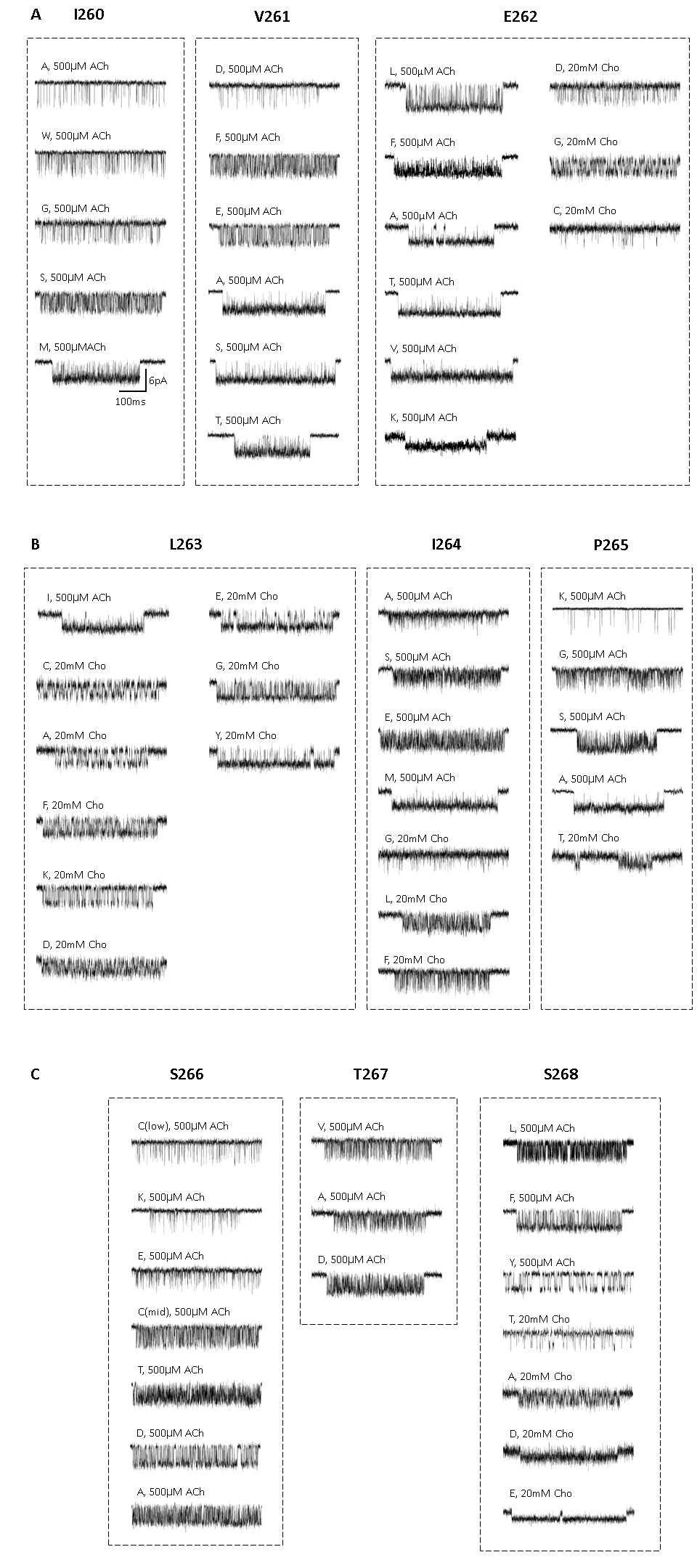

Supplement: Figure S1 — Single-channel current traces of various αM2 cap mutants (0.82 MB TIF) [file pone.0002515.s004.tif]
